# Connection between Periodontitis-Induced Low-Grade Endotoxemia and Systemic Diseases: Neutrophils as Protagonists and Targets

**DOI:** 10.3390/ijms22094647

**Published:** 2021-04-28

**Authors:** Ljubomir Vitkov, Luis E. Muñoz, Jasmin Knopf, Christine Schauer, Hannah Oberthaler, Bernd Minnich, Matthias Hannig, Martin Herrmann

**Affiliations:** 1 Vascular & Exercise Biology Unit, Department of Biosciences, University of Salzburg, 5020 Salzburg, Austria; lvitkov@yahoo.com (L.V.); hannah.oberthaler@sbg.ac.at (H.O.); bernd.minnich@sbg.ac.at (B.M.); 2 Clinic of Operative Dentistry, Periodontology and Preventive Dentistry, Saarland University, 66424 Homburg, Germany; 3 Department of Internal Medicine 3—Rheumatology and Immunology, Universitätsklinikum Erlangen, Friedrich-Alexander-University Erlangen-Nürnberg (FAU), 91052 Erlangen, Germany; luis.munoz@uk-erlangen.de (L.E.M.); jasmin.knopf@uk-erlangen.de (J.K.); christine.schauer@uk-erlangen.de (C.S.); martin.herrmann@uk-erlangen.de (M.H.)

**Keywords:** blood transmission of PAMPs, trained immunity, loss of tolerance, neutrophil hyper-responsiveness, neutrophil-driven damages

## Abstract

Periodontitis is considered a promoter of many systemic diseases, but the signaling pathways of this interconnection remain elusive. Recently, it became evident that certain microbial challenges promote a heightened response of myeloid cell populations to subsequent infections either with the same or other pathogens. This phenomenon involves changes in the cell epigenetic and transcription, and is referred to as ‘‘trained immunity’’. It acts via modulation of hematopoietic stem and progenitor cells (HSPCs). A main modulation driver is the sustained, persistent low-level transmission of lipopolysaccharide from the periodontal pocket into the peripheral blood. Subsequently, the neutrophil phenotype changes and neutrophils become hyper-responsive and prone to boosted formation of neutrophil extracellular traps (NET). Cytotoxic neutrophil proteases and histones are responsible for ulcer formations on the pocket epithelium, which foster bacteremia and endoxemia. The latter promote systemic low-grade inflammation (SLGI), a precondition for many systemic diseases and some of them, e.g., atherosclerosis, diabetes etc., can be triggered by SLGI alone. Either reverting the polarized neutrophils back to the homeostatic state or attenuation of neutrophil hyper-responsiveness in periodontitis might be an approach to diminish or even to prevent systemic diseases.

## 1. Introduction

A multitude of clinical findings demonstrated an unambiguous correlation between periodontitis and systemic diseases, like atherosclerosis, diabetes, and cardiovascular diseases [1]. The main link between systemic diseases and periodontitis is considered to be the systemic low-grade inflammation (SLGI); apparently a consequence of low-grade endoxemia (LGE) [2,3,4,5,6,7]. Endotoxins like lipopolysaccharide (LPS) are involved in the pathogenesis of many diseases such as atherosclerosis, obesity, chronic fatigue, metabolic syndrome, and many other inflammation-driven conditions. The detection of elevated plasma endotoxin levels provides evidence for each of these diseases [8]. Indeed, periodontitis [9,10] and even gingivitis [11] is characterized by LGE, which is considered a possible precondition for SLGI [12]. The exact signaling pathway responsible for triggering the systemic diseases caused by periodontitis-related LGE is not yet clarified. Understanding trained immunity progressed greatly in the last few years. Earlier, the idea of immune memory was reserved for the adaptive immunity. Growing in vivo and clinical evidences now indicate the ability of innate immunity to memorize bacterial challenges and to adjust its response to recurrent challenges, metabolically, epigenetically, and transcriptionally [13,14,15,16]. Certain microbial infections or vaccines promote a heightened response of myeloid cell populations to a subsequent infection with the same or even different pathogens. This process involves changes in cell transcription and is referred to as ‘‘trained immunity’’ [17,18]. The innate immunity fosters a sustained favorable response of myeloid cells to a secondary challenge, despite the short lifespan of some of these cells in circulation; trained immunity acts via modulation of hematopoietic stem and progenitor cells (HSPCs) [14].

This review discusses the accomplishment of LGE as the main link between periodontitis and systemic diseases. Long-term LGE in periodontitis is a consequence of disturbance of gingival barrier function, development of trained immunity, and emergence of hyper-responsive neutrophils. The trained immunity triggered by periodontitis-reliant LGE appears to be a crucial deteriorating factor in some systemic diseases, such as diabetes, atherosclerosis, and cardiovascular diseases [2,3,4,5,6,7]. This highlights the importance of periodontitis-dependent LGE and suggests the necessity of neutrophil calming as a new prophylactic approach to systemic diseases. Due to the huge domain of trained innate immunity (for references see [19]), we focused on the role of neutrophils in periodontitis, as they are responsible for the persistent periodontitis-reliant LGE, which leads to disbalanced trained immunity and systemic disease pathology.

## 2. Development of Trained Immunity

**Figure 1:**
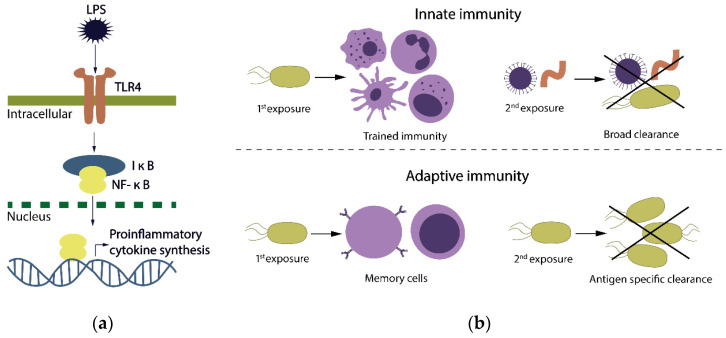
Concept of trained immunity. (**a**) The LPS signaling pathway via Toll-like receptor 4 (TLR4) and the nuclear factor kappa-light-chain-enhancer of activated B cells (NF-κB) constitutes a hallmark of both innate and adaptive immune activation. NF-κB is a protein complex controlling the transcription of DNA, cytokine production, cell survival. It orchestrates the immune response to infection. The activation of NF-κB is initiated by the signal-induced degradation of Nuclear factor of kappa light polypeptide gene enhancer in B-cells inhibitor (I-κB) proteins; and (**b**) comparison between innate and adaptive immunity. Innate and adaptive immunity provide broad and antigen-specific protection, respectively. (Top) innate immunity can provide protection against heterologous stimuli. Adaptive memory (bottom) is elicited with primary exposure to a specific antigen, and then, a secondary exposure and subsequent protection requires exposure to the same antigenic epitopes.

The concept of trained immunity describes the long-term functional reprogramming of the progenitors of innate immune cells evoked by exogenous or endogenous insults. Subsequently, this leads to an altered response of differentiated cells towards a second challenge, after returning to a non-activated state [19]. The secondary response to the subsequent non-specific stimulus can be altered in such a way that the cells respond more or less strongly, when compared to the primary response, conferring context- and time-adjusted responses [19]. Different stimuli, such as *β*-glucan [20], bacillus Calmette–Guérin (BCG vaccine) [16], and LPS [15] induce different programs of trained immunity. However, as periodontitis preferentially supplies LPS, we focus on the effects of LPS on innate immunity, in this review. HSPCs directly respond to LPS, e.g., via the toll-like receptor 4 (TLR4) [21,22] or indirectly through LPS-induced cytokines [23]. The pioneering transcription factor CCAAT-enhancer-binding protein *β* (C/EBP*β*) is induced by LPS in HSPCs and is required for the epigenetic chromatin marks and altered sensitivity of the associated genes to secondary challenges [15]. C/EBP*β* can act as a pioneer factor triggering chromatin opening of myeloid genes, without inducing their transcription [24]. It can pre-program chromatin accessibility for other transcription factors, leading to a facilitated response of DNA regulatory elements to stimulation [25]. LPS-exposed HSPCs become epigenetically primed for a myeloid lineage bias with the enhancers remaining more accessible than naive HSPCs. Furthermore, LPS-exposed HSPCs keep increased accessibility of numerous genes that predispose one to a more rapid activation of myeloid lineage commitment, in response to secondary stimulation [15]. As the innate memory responses depend solely on epigenetic remodeling, the trained immunity appears to lack specificity (Figure 1).

**Figure 2:**
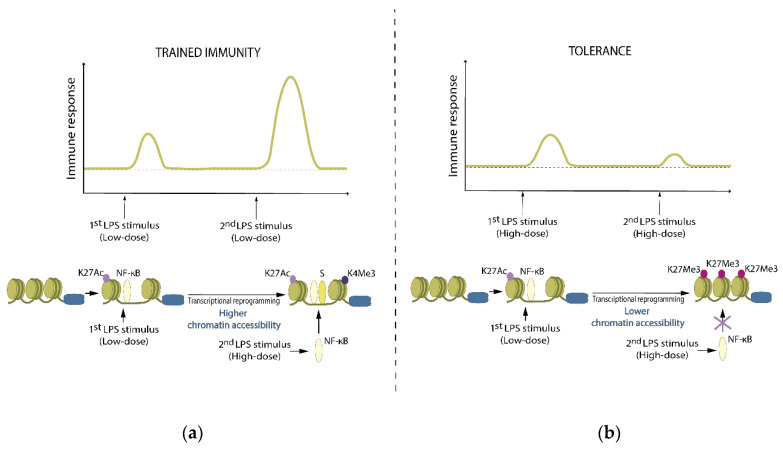
A scheme of a locus of inflammatory genes (olive-green) in myeloid cells showing (**a**) hyper-responsive adaptations elicited by a low-dose LPS exposure. The inflammatory stimulation with low-dose LPS triggers complex changes at the signaling, metabolic, or transcriptional level. These often result in the formation of H3K27ac (Ac) and an increased chromatin accessibility of the regulatory elements of the adapted genes. This favors the binding of transcription factors and gene expression after secondary exposure to another inflammatory stimulus (training) through the expression of H3K4me3. (**b**) Tolerization: Exposure to high-dose LPS elicits hypo-responsive adaptations. The deposition of repressive histone marks such as H3K27me3 (Me3) at regulatory elements of inflammatory genes prevents binding of transcription factors and efficient locus re-induction leading to tolerance. S: signal transducer and activator of transcription 1 (STAT1).

The molecular basis of the epigenetic modifications includes changes in chromatin organization at the level of the topologically associated domains, transcription of long non-coding RNAs, methylation and acetylation of genes involved in the innate immune responses, and reprogramming of cellular metabolism [19]. Most proinflammatory gene loci in the quiescent myeloid cells are in a repressed configuration [26], hindering access of the transcriptional machinery to the regulatory regions that drive the expression of inflammatory factors [27]. Two key epigenetic marks characterize trained immunity—the acetylation of histone 3 lysine 27 (H3K27ac) at distal enhancers (marked by histone 3 lysine 4 methylation (H3K4me1)) and the consolidation of histone 3 lysine 4 trimethylation (H3K4me3) at the promoters of stimulated genes [19] (Figure 2). The transmission of these marks through DNA replication and the cell cycle is robustly required to maintain trained immunity [28].

## 3. LGE Induces Systemic Diseases

LGE is a consequence of either metabolic disorders like metabolic endotoxemia or chronic infections like periodontitis. Prolonged LGE results in trained immunity, a long-lasting proinflammatory phenotype of leukocytes. LGE represents a strong risk factor for the occurrence of chronic inflammatory diseases such as diabetes, atherosclerosis, cardiovascular diseases, and others [29,30]. Here, we provide a concise overview of mechanisms responsible for initiations of systemic diseases by LGE.

Diabetes: Triggering of TLR4 activates proinflammatory pathways via activation of NF-κB and activator protein 1, which, in turn, promote production and release of multitudes of proinflammatory cytokines, including TNF-α and IL-6. This causes serine phosphorylation of the IRS-1, resulting in insulin resistance [31]. Thus, low doses of LPSs induce a biphasic change in glucose uptake in normal-weight volunteers. Insulin sensitivity enhances in the first few hours after injection [32] and significantly reduces later [33]. Doses of LPSs as low as 3 ng/kg are sufficient to induce a significant reduction of insulin sensitivity and increase circulation of insulin and glucose, 24 h after the LPS injection [33].

Cardiovascular diseases: LGE is associated with a significantly increased risk of cardiovascular diseases; however, their mutual relationship is still elusive. In cardiovascular disease patients, LGE triggers TLR4-mediated inflammatory responses, leading to SLGI [34]. A main representative of cardiovascular disease is the coronary artery disease resulting from a reduced blood flow to the heart muscle caused by luminal obstruction by atherosclerotic plaques in the arteries of the heart.

Atherosclerosis: Trained immunity is mechanistically linked to atherosclerosis [35]. LGE induces atherosclerotic plaque formation and progression of atherosclerotic lesions, and release of proinflammatory molecules from endothelial cells [36]. LPS appears to increase endothelial lipase, which was suggested to cause a reduction in HDL [37]. The latter supports the growth of atherosclerotic plaques [38]. A short-term treatment with LPS in a super-low-dose that mimics chronic infection elicits the polarization of monocytes into a sustained pro-inflammatory state. This is characterized by upregulation of lymphocyte antigen 6C, C-C chemokine receptor type 5, monocyte chemoattractant protein 1, and decreased expression levels of scavenger receptor class B type 1. It ultimately aggravates atherosclerosis [39]. LGE skews neutrophils into a non-resolving inflammatory state with elevated and reduced levels of inflammatory and homeostatic mediators, respectively [40]. These neutrophils are prone to NET formation [41,42], which further fosters atherosclerosis [43] and cardiovascular complications [44]. This implies that LGE drives many systemic diseases, predominantly acting through the over-activation of innate immunity.

## 4. Trained Immunity and Neutrophil Hyper-Responsiveness

While a (non-lethal) high-dose of LPS induces innate immune tolerance with an attenuated immune response to a second challenge, a super-low-dose of LPS induces trained immunity [39,45,46]. The latter is achieved via the reprogramming of HSPCs. Although trained immunity usually improves the host’s defense against subsequent pathogenic threats, in chronic inflammatory disease it might be maladaptive [19]. Thus, neutrophils and other leukocytes derived from HSPCs with trained immunity might develop in a distinct state of hyper-responsiveness; this might be responsible for the development of non-resolving inflammation. The nature of non-resolving inflammatory neutrophils is reflected in elevated reactive oxygen species, due to disrupted peroxisome homeostasis. This results in the skewed activation of oxidized calmodulin-dependent protein kinase II, downstream expression of LTB4, matrix-metalloproteinase (MMP) 9, miR24, and reduced ATF4/KLF2-mediated expression of resolving mediators, such as LRRC32 and miR-126 [40]. Indeed, neutrophil hyper-responsiveness is a hallmark of periodontitis [47,48,49,50]. Epigenetic reprogramming of HSPCs due to LGE makes neutrophils prone to exaggerated neutrophil extracellular trap (NET) formation [41,42]. NETs are evolutionarily conserved innate immunity structures produced by activated neutrophils, often in response to bacterial challenge. NETs have a backbone of DNA and contain neutrophil proteases, as well as other bactericidal agents [51,52,53,54]. Exaggerated NET formation causes crevicular ulcers (see Section 6.2), subsequent bacteremia and LGE. Diabetes can induce late-onset periodontitis [55,56], as it promotes neutrophils to form NETs [57]. The boosted NET formation is a part of trained immunity and is epigenetically reprogrammed through the acetylation of histone 4 in neutrophils [41,42]. Subsequently, the transcriptomes of hyper-responsive neutrophils in periodontitis are altered [58]. Due to trained immunity, the neutrophil hyper-responsiveness remains in edentulous patients with a history of periodontitis [47,48,49,50], despite the disappearance of bacteremia after teeth exfoliation [59]. The fact that the hyper-responsiveness of neutrophils from patients with periodontitis is evident in vitro, where the influence of adaptive immunity is excluded, cannot be explained in other way than by trained immunity. However, neutrophils are comprised of certain subsets [60], so they might have different effects on periodontal and systemic disease (SD) pathology. In vivo, many components modulate neutrophil response, e.g., innate immunity, adaptive immunity, environment, etc. Besides the innate immunity mechanisms responsible for deactivating LPS (see Section 6), many adaptive immunity mechanisms are able to counteract the neutrophil hyper-reactivity in vivo. Therefore, patients with periodontitis are de facto immunized with the LPS of periodontal pathogens [61]. Normal human serum contains polyreactive “natural” antibodies that bind LPS [62]. LPS antibodies to periodontal pathogens [63] are elevated in periodontal patients, as compared to subjects with a healthy periodontium. Regulatory T cells (Treg)/neutrophil interactions in inflammatory and autoimmune diseases play a crucial role for the neutrophil regulation and suppression [64]. The pro-inflammatory function of neutrophils in the promotion and pathogenesis of several autoimmune diseases like vasculitis [65], rheumatoid arthritis [66] etc., was reported. Defective Treg function were demonstrated in all these diseases, and Treg therapy ameliorated them [67]. Reduced Treg numbers in mice leads to exaggerated neutrophil activity resulting in mortality in endotoxic shock [68]. Tregs regulate survival and activity of human and murine neutrophils, and co-cultures of Tregs and neutrophils increases neutrophil apoptosis [68]. In addition, LPS-activated Tregs inhibit neutrophil functions [69,70,71] and even promote their apoptosis [69]. Thus, despite the periodontitis-induced hyper-responsibility, neutrophils cannot autonomously determine the entire immune response since it is orchestrated by the adaptive immunity. Therefore, it is not surprising that periodontitis is not ever associated to LGE-related SDs. Due to the huge complexity of the interactions between innate immunity and adaptive immunity, it is impossible to currently draw conclusions regarding the extent to which the periodontitis-related trained immunity define LGE-related SDs. Future investigations might show to which extent this connection is clinically relevant.

## 5. Correlation between Periodontitis and LGE-Related SDs, the Role of Periodontal Pathogens

Increasing the periodontitis severity carries a proportionally higher risk of coronary artery disease [72]. The relationship between periodontitis and coronary artery calcification indicates that periodontitis correlates positively and linearly with the presence of coronary artery calcification [73]. A relationship between periodontitis and aortic vascular inflammation exists, which in turn is a surrogate of coronary disease [74]. Live *Porphyromonas gingivalis* and *Aggregatibacter actinomycetemcomitans* were isolated from atheromatous lesions [75,76]. Furthermore, periodontal pathogens have the ability to induce atherosclerosis in animal models [77,78,79].

Many reports clearly demonstrated the association between diabetes and periodontal disease in both animals and humans. LGE might induce diabetes predominantly due to the bacterial burden of periodontitis (see Section 3). The resulting proinflammatory cytokines cause insulin resistance [31,33,80]. In addition, *P. gingivalis* contributes to the development of insulin resistance via an impaired adaptive immune response [81]. Interestingly, treating the periodontal disease reduced glycated hemoglobin in diabetic patients [82,83,84].

The high co-incidence between periodontitis and LGE-related SDs, e.g., cardiovascular diseases, diabetes etc., as well as the ability to induce some SDs via periodontitis in animal model, leads to the question as to how periodontitis might yield an initial ignition of LGE-related SDs? Three periodontitis aspects might be considered:

(i) Transient supply of LPS as bacterial fragments and whole bacteria in periodontitis [85,86,87,88]. As an enduring LPS supplier, this aspect of periodontitis appears to be a crucial factor in triggering the LGE-relied SDs.
(ii) Trained immunity marked by periodontitis-induced neutrophil hyper-responsiveness [47,48,49,50]. This aspect might be responsible for the gingiva ulceration, and hence might act as a promoter of both LGE and bacteremia (see Section 6.2.);
(iii) An independent settlement of periodontal pathogens in the atherosclerotic plaques [79,89,90,91]. The periodontal pathogens appear able to induce and promote atherosclerosis in humans [75].

## 6. Endotoxemia and LPS Metabolism

LGE can be induced in many different ways. However, the mechanisms of LPS transition into the blood of patients with periodontitis remain elusive. The enteral LPS content is the main reservoir of LPS within the human body [92], but the intestinal barrier function prevents LPS transmission into the blood of healthy individuals. (I) The mucin layer limits the access of LPS to the epithelium. (II) A multitude of cationic antimicrobial host proteins binds the negatively charged LPS [93]. (III) Intestinal alkaline phosphatase detoxifies LPS by the cleavage of the phosphate groups in position 1 and 4 of lipid A. The dephosphorylated LPS does not trigger TLR4 signaling [94]. (IV) The LPS-binding protein (LBP) forms a large LPS–LBP complex, with a reduced ability to trigger TLR4 signaling [95]. (V) LPS that enters the bloodstream, i.e., into the portal vein, is detoxified in the liver and excreted into the bile. Taken together, intestinal LPS does not abundantly transit into the peripheral blood of individuals with healthy intestines [92]. Alterations of the intestinal microbiota (dysbiosis) lead to increased intestinal permeability and translocation of LPS to the blood circulation. This disorder is referred to as metabolic endotoxemia ME. Dysbiosis also leads to the production of trimethylamine-N-oxide, a gut bacterial metabolite discussed as a new risk factor for the development of cardiovascular diseases [96]. Hence the question arises as to how LPS of periodontitis origin transmits into the blood of intestinally heathy individuals? LPS is able to even penetrate healthy gingival epithelium in very small quantities [97], as it is detoxified by blood proteins, blood enzymes, and neutrophil-derived enzymes [98]. This, consequently, does not contribute to the elevation of the blood LPS level. However, increased blood plasma LPS levels were reported both in late [9] and early-onset periodontitis [10] and even transient bacteremia during daily hygienic procedures [86,87,88]. These clinical findings imply three key points:

- LPS penetration occurs via the inflamed periodontal pocket.
- LPS penetration might be to, a large part, a consequence of transient bacteremia.
- Intermittent pressure on the pocket epithelium appears to be crucial for bacteremia and subsequent endotoxemia.

### 6.1. LPS Penetrates the Periodontal Pocket

Increased blood plasma levels of LPS were reported in gingivitis [11] and characterize periodontitis [9,10]. The source of the LPS is the subgingival plaque (biofilm). A part of the LPS unleashed into the crevice is bound by blood proteins, neutrophil cationic antimicrobial proteins [99,100], neutrophil enzymes, and by LPS antibodies. Thus, a diverse repertoire of inactivation mechanisms modulates the ability of LPS to activate neutrophils via TLR4 [101]. The concentrations of soluble CD14 (sCD14) [102], LPS-binding protein [103], and antibodies to LPS of periodontal pathogens [63] are elevated in periodontal patients, as compared to subjects harboring a healthy periodontium.

In vitro *P. gingivalis* induces an increase of the gingival permeability through the secreted gingipains, enabling macro-molecules to diffuse through the tight junctions [104]. LPS within polar, aqueous fluid forms aggregates several tens of nanometers in size [105]. Moreover, LPS is discharged by bacteria in the form of outer membrane vesicles in a similar size range [106], or as fragments of the outer bacterial membrane in cases of bacterial death. The periodontal crevice, where the dental plaque grows, is confined between the dental root surface and the crevicular gingival epithelium. The latter continuously produces blood transudate denoted gingival crevicular fluid (GCF) [107,108]. The incessant GCF flow that drains off the LPS. *P. gingivalis* is not a prerequisite for periodontitis. The host inactivation mechanisms operate on mucosal surfaces and in tissues, lymph, and blood, and might profoundly influence LPS bioactivity in vivo [8,109].

LPS is first encountered by plasma proteins and the enzymes of the GCF, which is just blood plasma transudate. Alkaline phosphatase is abundant in GCF [110,111] and inactivates LPS by dephosphorylation [112]. Acyloxyacyl hydrolase, produced by crevicular neutrophils [113] deactivates LPS through the removal of the secondary acyl chains from lipid A. Even though LPS penetrates the epithelium in gingivitis [11], it is partially bound by the blood plasma components like HDL and LDL [114,115,116]; sCD14 binds and transfers LPS from leukocytes to HDL [116,117]. The bactericidal-permeability protein binds the LPS produced by many Gram-negative bacteria and blocks lipid A bioactivity [118,119]; Gelsolin, a highly conserved plasma protein, binds and neutralizes LPS [120]; human serum from healthy individuals contains polyreactive “natural” antibodies that bind and neutralize LPS antigens [62].

Gingivitis is a transitory inflammation and is not yet connected to trained immunity. Sustained untreated chronic gingivitis leads to pocket formation and consequently to transition into periodontitis. Due to the features of the periodontal pocket, which is a pathological formation, periodontitis must be considered a non-resolving chronic inflammation [121]. It is characterized by the formation of crevicular NETs, [122,123], multiple gingival micro-ulcerations [124] and bacteremia [85,86,87,88]. The periodontitis-related bacteremia is responsible for the direct contact of LPS with HSPCs.

### 6.2. Ulcerations Circumvent the Epithelial Barrier in Periodontitis

The exaggerated NET formation in the crevice drastically changes the situation, as NETs damage the epithelium, such that both bacteria and their pathogen-associated molecular pattern (PAMPs) overcome the epithelial barrier. On one hand, the topically-liberated LPS of the gingiva binds host proteins (LPS, antibodies as well as enzymes), and on the other hand, the periodontitis-related endotoxemia appears to be concomitant with bacteremia. Thus, the question arises, whether periodontitis-related endoxemia is just a consequence of the transitory bacteremia? Bacteremia requires discontinuations of crevicular epithelium in order to transmit the whole bacteria into connective tissue. Indeed, the crevicular epithelium is characterized by multiple micro-ulcerations [124]. Epithelial ulcerations enable the invasion of microorganisms and the unrestricted penetration of their PAMPs into the connective tissue, and thus aggravate the course of periodontitis. The influx of neutrophil and NET-derived proteases into the connective tissue facilitates bacterial spread. Therefore, both in late-onset [125,126] and experimental periodontitis [59], crevicular NETs are unable to completely prevent periodontal pathogen dissemination into connective tissues and peripheral blood. The latter becomes evident as bacteremia (number of species and positive cultures), which increases with the severity of the gingival inflammation [127]. The periodontitis-reliant bacteremia disappears after teeth exfoliation [59]. Epithelial ulcerations are of interest insofar as they represent the entry points for oral pathogens and their PAMPs, especially LPS. This results in bacteremia and endotoxemia. Epithelial disruptions such as insect bites or minor skin scratches [128] and gingival micro-ulcerations [86,87,88] are also accompanied by transient bacteremia and endotoxemia. In experimental periodontitis, disseminated oral pathogens are regularly observed within both liver and spleen, but not in mice after complete teeth loss [59]. Ulcers of oral epithelium other than the crevicular epithelium are very common, but they are mostly due to hereditary and environmental factors. Only in cases of acute necrotizing gingivitis, syphilis and tuberculosis are caused by bacteria [129,130]. Indeed, oral pathogens penetrate crevicular epithelium either by bacterial internalization [131] or mechanically (yeasts) [132] without the formation of ulcers. Thus, bacterial pathogens appear to not directly cause gingival ulcerations. Otherwise, hyper-responsive neutrophils and in particular exaggerated NET formation are able to damage mucosal epithelium. The main task of NETs is to limit the bacterial spread [51], but exaggerated NET formation is destructive [133]. Exaggerated NET formation in the periodontal crevice [99] results in oversupply of neutrophil proteases [134,135,136]. These proteases damage the epithelial basal lamina if the NETs are not aggregated [137] and laminin-332 breakdown products foster neutrophil recruitment, which damage epithelia and subsequently weaken the epithelial barrier [138]. NET-derived components such as histones [139,140,141,142] and myeloperoxidase (MPO) [140] are cytotoxic to epithelial cells; neutrophil proteases damage and even kill the epithelial cells. Some periodontal pathogens might even promote epithelial ulceration by stimulating the release of neutrophil elastase (NE) [143]. Indeed, increased GCF levels of laminin-332 [144,145,146] and neutrophil proteases [134,135,136] correlate with the epithelial ulceration in periodontitis [121,124]. NETs also cause epitheliopathy via Oncostatin M [147]. Gingival epithelium becomes ulcerated in late-onset [121] and in experimental periodontitis [148,149]. Obviously, epithelial ulcerations enable the contact between connective tissue matrices and NET-bound NE and MMPs, which are much more aggressive than the soluble enzymes [138]. High NET concentrations reportedly suppress keratinocyte proliferation, delay wound closure [57,150], and hence prolong the persistence of ulcers. However, aggNETs proteolytically inactivate several soluble pro-inflammatory mediators. Except for limited histological examinations, the ulcers of crevicular epithelium are not extensively studied and their role for systemic diseases remains elusive.

### 6.3. The Pocket Pump

The continuously secreted GCF [107,108] produces GCF flow, which drains off the disseminated bacteria and PAMPs. However, the retention of GCF in the pocket rises with deepening the periodontal pocket and increasing the GCF viscosity, particularly due to suppuration [151]. Intermittent pressure on the gingiva during mastication and dental hygiene is an underestimated problem and publications on this topic are scarce. However, clinicians should never forget to instruct dental patients after intra-pocket application of medications [152] and guided bone regeneration [153] to restrain from chewing. During mastication and dental hygiene procedures, the food bolus or the tooth brush, exerts intermittent pressure onto the oral gingiva, whereby the crevicular content (GCF and dispersed bacteria) is also pressed towards the pocket epithelium. In the ulcers, where matrices of the connective tissue are partly dissolved by exaggerated NETs, bacteria might be deeply inserted and even reach the venules. The high tendency of the venules to bleed in periodontitis and the transitory bacteremia/endotoxemia during mastication [85], tooth brushing and flossing [86,87,88], suggests such a portal of entry for pocket bacteria. Thus, the periodontal pocket might be considered to be a pump, which presses bacteria and their metabolic products into gingival connective tissues. From there they are pushed by intermittent mastication pressure into the gingival venules and reach the blood circulation via vena cava superior, conditioning both periodontitis-related bacteremia and endotoxemia. Thus, endotoxemia might be largely a consequence of transient bacteremia and lysis of the blood-borne bacteria, as the circulating LPS is continuously detoxified by the liver [92].

Intermittent pressure on the pocket epithelium and consequently within the crevice appears to be crucial for bacteremia and endotoxemia. Despite the fact that the phenomenon of intermittent pressure is familiar, up to now it was considered clinically irrelevant and is not yet examined. Thus, its examination and the exact mechanism of bacterial penetration through the gingiva into blood are indispensable to enable further progress in the periodontal pathology.

## 7. Targeting Neutrophils in the Prophylaxis of Systemic Diseases and Periodontitis

It is empirically known that the treatment of periodontitis attenuates systemic inflammatory diseases [154]. A main reason for this appears to be the reduction of the periodontitis-related LGE. The latter skews neutrophils into a non-resolving inflammatory state with elevated the levels of the inflammatory mediators dectin-1, MMP9, and leukotriene B4 (LTB4), and reduced the levels of the homeostatic/anti-inflammatory mediator leucine-rich repeat containing 32 (LRRC32), transforming growth factor–*β*, and ferroportin (FPN) [40]. The reduction of LGE by the treatment of periodontitis attenuates the LPS effects on neutrophils and might be a favorable approach for the mitigation of systemic diseases. Treatment options of periodontitis and their limitations are well-known in clinical periodontology. Therefore, here we focus on mitigating the inflammatory effects of LPS on neutrophils. In general, two strategies are conceivable—(i) reverting the polarized neutrophils back to the homeostatic state or (ii) diminishing the exaggerated crevicular NET formation.

### 7.1. Reverting the Polarized Neutrophils back to the Homeostatic State

A potential candidate is 4-phenylbutyrate (4-PBA), known to restore peroxisome homeostasis in other cells [155,156,157,158]. Recently, the application of 4-PBA was demonstrated to be a novel and effective strategy to revert polarized neutrophils back to their homeostatic state [40]. Thus, 4-PBA restores peroxisome–lysosome fusion in neutrophils and reduces the LPS-mediated elevation of neutrophil-derived ROS. In in vitro cultures of neutrophils, 4-PBA effectively reduced the induction of oxidized calmodulin-dependent protein kinase II, LTB4, MPO, dectin-1, CD11b, and miR-24 through a super-low-dose LPS and also restored the expression of ATF4, FPN, LRRC32, and miR-126 that was suppressed by LPS [40]. This functional rejuvenation of homeostatic neutrophils by 4-PBA treatment might reduce the pathogenesis of experimental atherosclerosis [40] and of related cardiovascular complications.

### 7.2. Diminishing the Exaggerated Crevicular NETs

Despite the ability of aggNETs to prevent and even resolve inflammation on mucosal surfaces [109] and other tissues [159,160], they also cause an increase in the viscosity of GCF [122]. This hinders the GCF evacuation from deep periodontal pockets and potentially drives the formation of periodontal abscesses [151]. Diminishing the exaggerated NET formation, which appears to be a part of some cases of trained immunity [41,42] by inhibitors of peptidylarginine deiminases 4 (PADI4), can reduce the periodontitis-related LGE and subsequently attenuate systemic diseases. As NETs directly promote atherosclerosis, the use of specific PADI4-inhibitors, in order to suppress the PADI4-dependent NET formation, might have therapeutic benefits in atherosclerosis [43] and cardiovascular complications [44]. In humans, metformin treatment reduces the concentrations of NET components independent of glucose control. In vitro this effect was related to the inhibitory effect exerted on the protein kinase C-nicotinamide adenine dinucleotide phosphate oxidase pathway [161]. Using topical treatment in periodontitis might prevent the systemic side effects of neutrophil inhibitors.

Taken together, both strategies aim at reducing the periodontitis-related LGE. Future treatment strategies are needed to diminish neutrophil hyper-responsiveness and, in particular, their propensity for NET formation, as neutrophil hyper-responsiveness fosters both non-resolving inflammation and LPS penetration through the gingival epithelium.

## 8. Conclusions

LGE is blamed for many systemic diseases, like diabetes, atherosclerosis, cardiovascular diseases, and others. Periodontitis, similar to other chronic infections, causes LGE and hence might foster systemic diseases. LGE might turn innate immunity into a state of trained immunity with the hyper-responsive neutrophils prone to exaggerated NET formation. The latter is responsible on one hand for gingival ulcerations and subsequent bacteremia/endotoxemia, and on the other hand for damages of blood vessels and other host tissues observed in systemic diseases. Attenuation of periodontitis reduces the periodontitis-related LGE and is prone to ameliorate the LGE-related systemic diseases. Modulation of innate immunity might be a promising approach to diminish systemic inflammatory diseases and in the treatment of periodontitis.

## Data Availability

Not applicable.

## Abbreviations

FPN	ferroportin
GCF	gingival crevicular fluid
HDL	high density lipoprotein
HSPC	hematopoietic stem and progenitor cells
IL	interleukin
LBP	LPS-binding protein
LDL	low density lipoprotein
LGE	low-grade endoxemia
LPS	lipopolysaccharide
LRR	leucine-rich repeat containing
MDPI	multidisciplinary Digital Publishing Institute
MMP	matrix metalloprotease
NETs	neutrophil extracellular traps
SDs	systemic diseases
PADI4	peptidylarginine deiminases 4
PAMPs	pathogen associated molecular patterns
PMN	polymorphonuclear cells
SLGI	systemic low-grade inflammation
TLR	toll-like receptor
TNF	tumor necrosis factor
Treg	Regulatory T cells
